# Successful treatment of esophageal perforation due to black esophagus (acute esophageal necrosis): a case report

**DOI:** 10.1186/s44215-023-00115-5

**Published:** 2023-11-16

**Authors:** Takahiro Ochiai, Shinsuke Takeno, Fumiaki Kawano, Kousei Tashiro, Atsushi Nanashima, Ryo Tsuzuki, Kouichi Doi

**Affiliations:** 1 Department of Surgery, University of Miyazaki Faculty of Medicine, Miyazaki, 889-1692 Japan; 2 Department of Diagnostic Pathology, University of Miyazaki Faculty of Medicine, Miyazaki, Japan; 3 Department of Surgery, Miyazaki Prefectural Nobeoka Hospital, Miyazaki, Japan

**Keywords:** Black esophagus, Acute esophageal necrosis, Minimally invasive esophagectomy, Duodenal ulcer perforation

## Abstract

**Background:**

Black esophagus, or acute esophageal necrosis (AEN), is a rare disease with a poor prognosis in which the esophageal mucosa is black in color. We report a case of esophageal perforation due to AEN that was successfully treated.

**Case presentation:**

An 88-year-old woman presented to her local hospital with the chief complaint of abdominal pain. Endoscopic and radiological examinations revealed esophageal perforation due to AEN and duodenal perforation. Omental patch repair was performed for the esophageal perforation, and the duodenal ulcer perforation was treated by simple closure and gastrojejunostomy at the local hospital. Following refractory esophageal and duodenal perforations after the initial surgery, the patient was transferred to our hospital where emergent surgery was performed, comprising thoracoscopic esophagectomy, cervical esophagostomy, T-tube drainage, and omentopexy for duodenal perforation, as well as thoracic and abdominal drainage. Mucosal regeneration of the esophagostomy was confirmed endoscopically on postoperative day 19.

**Conclusions:**

Appropriate surgical intervention may be an optimal option in patients with esophageal perforation due to AEN.

## Background

Black esophagus, or acute esophageal necrosis (AEN), defined as necrosis of the esophageal mucosa, is a rare disease with a prevalence of 0.1–0.28% [1]. It has been reported that the typical patient is an older male with general debilitation and multiple comorbidities, presenting with hematemesis or melena [2]. The incidence of esophageal perforation is 6.8%, which requires urgent surgical intervention [3, 4]. We present the case of an 88-year-old woman with AEN. The patient was rescued by emergent surgery that comprised thoracoscopic esophagectomy, cervical esophagostomy, T-tube drainage, and omentopexy for AEN and duodenal perforation, as well as thoracic and abdominal drainage.

## Case presentation

**Fig. 1:**
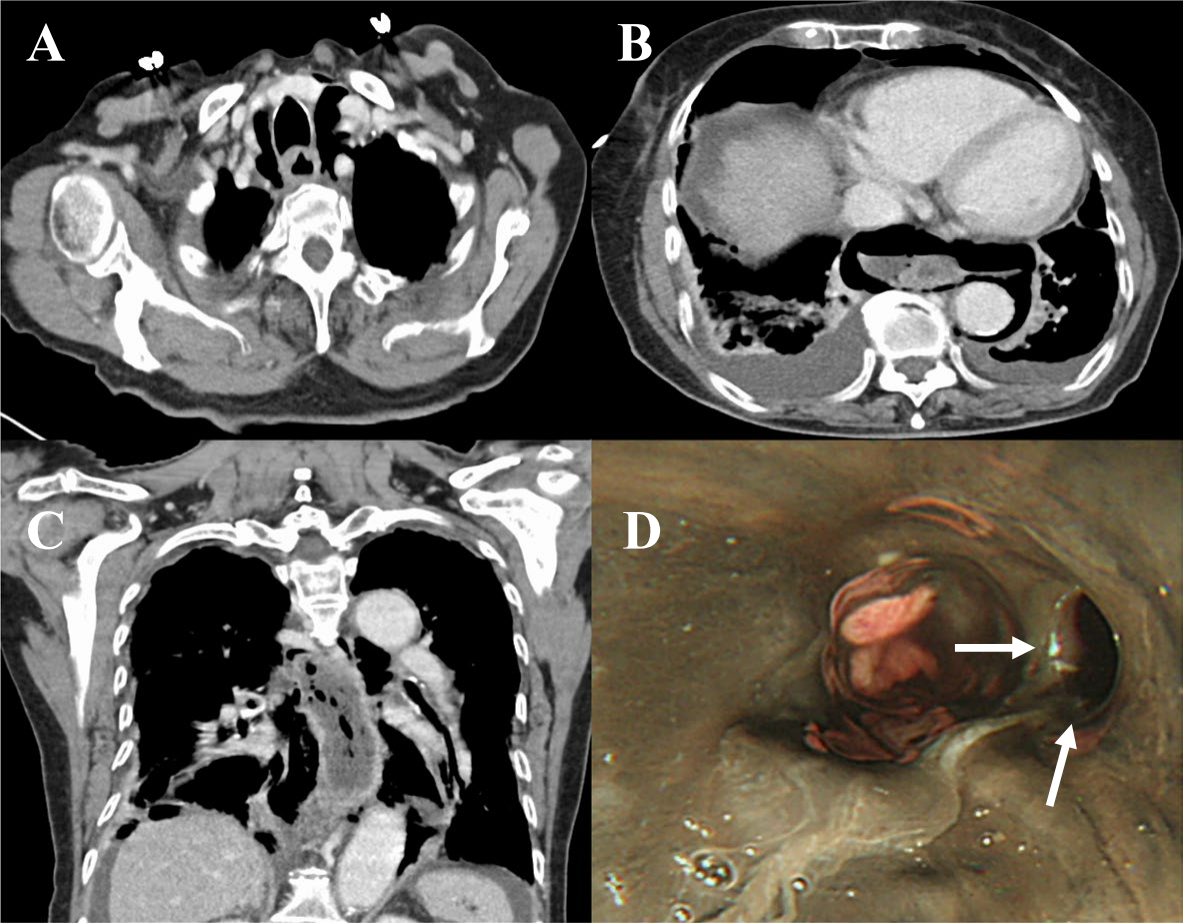
Preoperative CT and intraoperative endoscopy. **A**–**C** CT shows free air in the broad area from the upper mediastinum to the upper abdomen, suggesting lower esophageal perforation. **D** Intraoperative endoscopy shows mucosal necrosis affecting the entire esophagus and perforation in the lower esophagus (white arrows)

**Fig. 2:**
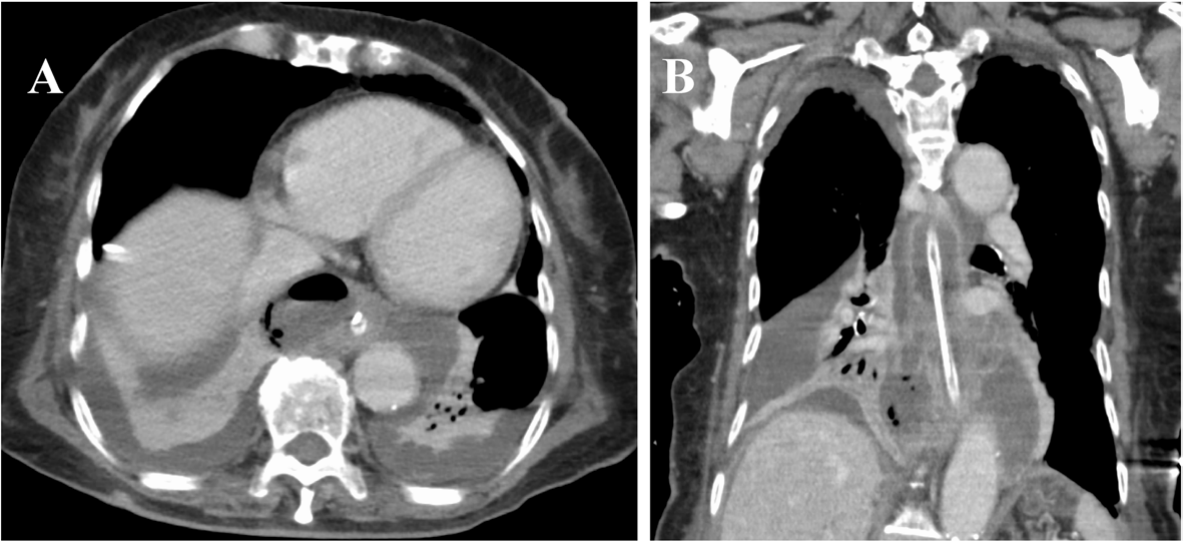
Postoperative CT. **A** and **B** CT shows fluid collection around the lower esophagus and poor contrast on the esophageal wall, suggesting poor drainage and residual perforation

An 88-year-old woman with a history of duodenal ulcer was transported by ambulance to her local hospital with the chief complaint of abdominal pain over the preceding 2 days. She had no underlying comorbidities and was not on any medications. She had no hematemesis, nausea, or vomiting but was in septic shock, with a systolic blood pressure of 78 mm Hg, heart rate of 124 beats/min, and temperature of 37.3 °C. Laboratory findings showed a white blood cell count of 4100/μL; C-reactive protein of 9.69 mg/dL; arterial blood gas with a pH of 7.25, PCO_2_ of 35.4 mmHg, PO_2_ of 75.6 mmHg, and HCO_3_ of 16.0 mEq/L; and base excess of −10.7 mEq/L. CT showed free air in the broad area from the upper mediastinum to the upper abdomen, suggesting lower esophageal perforation (Fig. 1A–C). Ascites was noted, and intestinal blood flow was unremarkable. No vascular malformations, vasodilatations, or aneurysms were observed in the celiac artery or other major vessels. An esophagectomy was not possible due to the absence of an esophageal surgeon, and an emergent omental patch repair by the trans-hiatal approach was planned for the esophageal perforation. Operative findings showed an ulcer perforation with a diameter greater than 5 cm in the anterior wall of the first portion of the duodenum in addition to esophageal perforation. The duodenal perforation was simply sutured, the duodenum was transected from the stomach, and gastrojejunostomy and jejunostomy were performed to facilitate enteral nutrition after the omental patch for the esophageal perforation. At surgery, the esophageal mucosa was black in color, and intraoperative endoscopic examination showed mucosal necrosis of the entire esophagus (Fig. 1D). The patient’s postoperative hemodynamic status was stable, but CT findings on postoperative day 2 showed fluid collection around the lower esophagus and poor contrast on the esophageal wall, suggesting poor drainage and residual perforation (Fig. 2A, B).

**Fig. 3:**
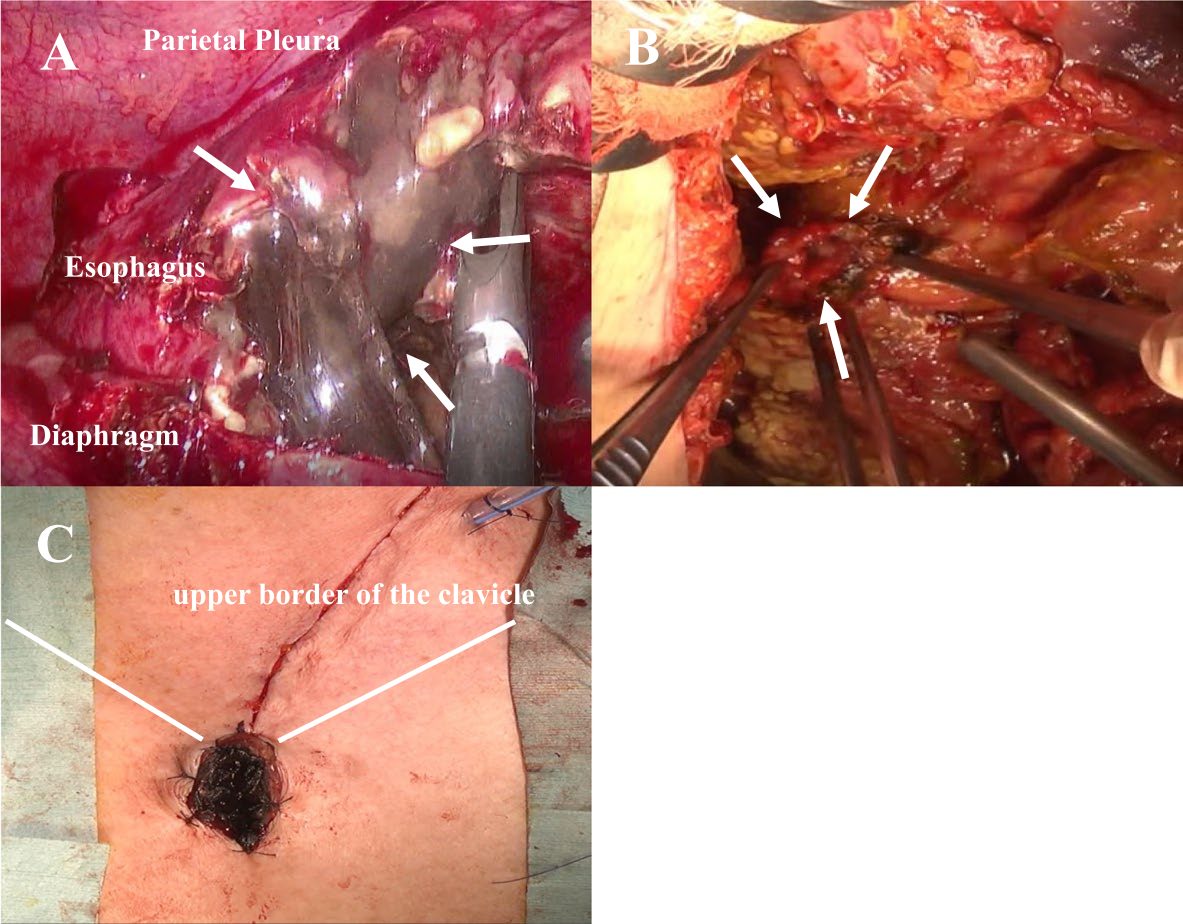
Operative findings. **A** Perforation greater than 3 cm in the lower thoracic esophagus (white arrows) remains, and the esophageal mucosa changes to black at surgery. **B** The duodenal suture failure was observed (white arrows). **C** Esophagostomy is made on the manubrium to facilitate additional resection of the necrotic esophagus

Therefore, the patient was transferred to our hospital for further treatment on the third day after the initial rescue surgery. Thoracoscopic subtotal esophagectomy was planned for the AEN. At surgery, there was a remaining perforation greater than 3 cm in the lower thoracic esophagus, and the esophageal mucosa was black in color (Fig. 3A). The esophagus was resected at the level of the tracheal bifurcation; however, due to the incomplete omental patch repair performed for esophageal perforation during the initial surgery, there was difficulty viewing the perforation in the lower mediastinum. In addition, an omental patch repair was performed for esophageal perforation at the initial surgery, and resection by thoracic manipulation was judged to be difficult. The abdomen was subsequently opened, and the lower esophagus was dissected transhiatally and resected along with the upper portion of the stomach.

Since duodenal suture failure was observed (Fig. 3B), a T-tube was then placed into the duodenum, with omentopexy using omentum fed by the left gastroepiploic artery. Because the entire esophageal mucosa had turned black, an esophagostomy was performed on the esophagus at the level of the manubrium to facilitate additional resection of the necrotic esophagus (Fig. 3C). The operation time was 4 h and 47 min, and blood loss was 600 mL.

**Fig. 4:**
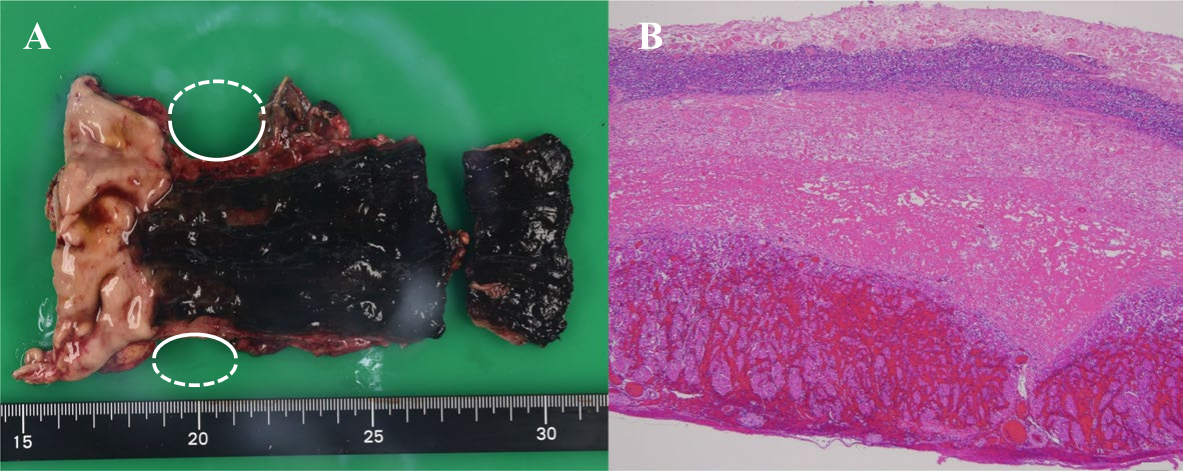
Gross and histopathological appearances of the resected specimen. **A** Macroscopic view of the resected specimen, showing perforation of the lower esophagus (white ellipses). The esophageal mucosa has totally turned black, and the gastric mucosa is intact. **B** Histopathologically, extensive necrosis is observed from the mucosal layer to the submucosal layer, extending partially to the muscularis propria. Neutrophilic infiltration is prominent in the lamina propria and extends to the adventitia

Macroscopically, the resected specimen of esophageal mucosa was completely black, and the gastric mucosa was intact (Fig. 4A). Extensive necrosis was observed from the mucosal to submucosal layers, extending partially to the muscularis propria histopathologically. Neutrophilic infiltration was prominent in the lamina propria and extended to the adventitia (Fig. 4B).

**Fig. 5:**
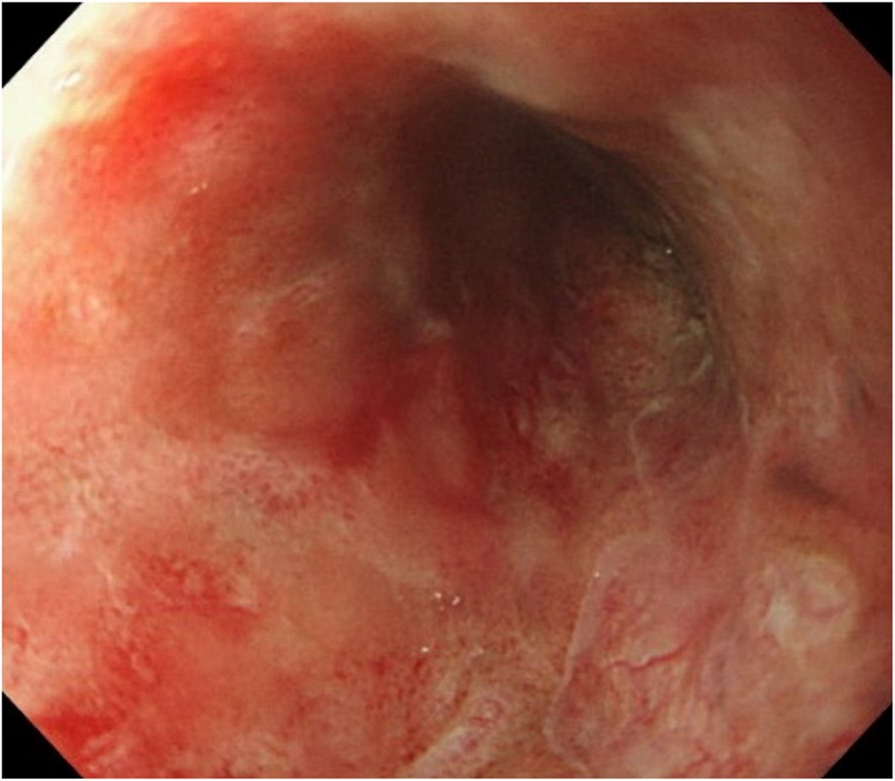
Postoperative endoscopic findings. Endoscopic examination shows regeneration of the esophageal mucosa on postoperative day 19

The patient’s condition stabilized, and endoscopic examination showed regeneration of the esophageal mucosa on postoperative day 19 (Fig. 5). The patient was transferred to the initial hospital for rehabilitation on postoperative day 20.

A two-stage reconstruction was planned, but due to family wishes, the decision was made not to perform reconstructive surgery.

## Discussion and conclusions

AEN is a rare disease with poor prognosis. In the 28-year period between when it was first reported by Goldenberg in 1990 until 2018, only 160 cases have been reported [5, 6]. It has been reported that in more than 90% of cases, AEN develops in the distal esophagus, and only in 33.7% of cases, such as the present case, does it reach the upper esophagus [6].

Its etiology is multifactorial, with the pathophysiology generally attributed to a combination of multiple mechanisms that include esophageal ischemia, reflux injury due to gastric content inflow, and impaired mucosal reparative mechanisms associated with frail physical states [2–4]. The common risk factors include hemodynamic impairment, lactic acid and diabetic ketoacidosis, stroke, dyslipidemia, alcoholism, trauma, local esophageal infection, surgery, postoperative conditions, drugs, chronic lung disease, and acute gastric outlet obstruction [2–4]. In the present case, it is thought that AEN probably resulted from circulatory failure due to septic shock caused by perforation of the duodenal ulcer.

There are scattered cases of AEN with duodenal involvement, as in the present patient. To the best of our knowledge, 37 cases associated with duodenal involvement have been published [1, 7–15].

It has been reported that the first and second parts of the duodenum receive their blood supply from the celiac artery, and that the common blood supplies of the distal esophagus and the first part of the duodenum suggest a pathogenetic association between AEN and duodenal ulcers [15]. However, because the esophagus has a rich segmental and intramural blood supply [7], it is difficult to understand how the two can be related on the basis of hypoperfusion of the celiac artery. A problem with celiac artery blood supply is an unlikely explanation for the present case as the entire esophageal mucosa was necrotic and the upper esophagus and middle esophagus were not supplied by the celiac artery. No vascular malformations, vasodilatations, or aneurysms associated with the AEN were found in the present case.

Dimitrios et al. performed a systematic review of the literature published between 1990 and 2018. Overall mortality associated with AEN was approximately 30%. Including complications other than perforation, 24.6% of patients required surgical or endoscopic intervention, and 75.4% were treated conservatively such as by fasting and the administration of proton-pump inhibitors [6].

Complications of AEN requiring surgical and endoscopic intervention have been reported as including strictures (10.2%), mediastinitis/abscess (5.7%), and perforation (6.8%) [3, 16]. Although severe cases of perforation in AEN are rare, it is a life-threatening complication that requires emergency surgery.

In a report of 130 cases of AEN compiled by Abdullah et al., there were 6 patients with perforation, 5 of whom underwent surgery and 1 of whom deteriorated rapidly following esophageal perforation, subsequently dying without undergoing surgery. Of the five surgical cases, four underwent esophagectomy [17]. Successful conservative treatment of esophageal perforation associated with AEN has not been reported. Given the compromised state of the patient due to AEN, esophagectomy is necessary to control perforation and infection. In the present case, the patient had sepsis due to esophageal perforation, and the AEN involved the entire esophagus. There was a risk of further perforation of the esophagus outside of the perforated area, and a subtotal esophagectomy seemed to be an appropriate decision.

In the present case, a massive surgical intervention consisting of esophagectomy for the esophageal perforation and T-tube drainage for the duodenal perforation was performed that led to survival of the patient.

In conclusion, a rare case of successful treatment of a rare case of potentially fatal AEN was reported. Although the prognosis in AEN remains poor, appropriate surgical intervention may rescue the patient. Further analysis is necessary to clarify the pathophysiology and establish a therapeutic strategy for this rare disease.

## Data Availability

The datasets used and/or analyzed during the current study are available from the corresponding author on reasonable request.

## Abbreviations

CT: Computed tomography
AEN: Acute esophageal necrosis
